# Kentucky State Medical Society

**Published:** 1874-05

**Authors:** 


### KENTUCKY STATE MEDICAL SOCIETY.

Our correspondent "B." sends us from Louisville, an interesting abstract of the proceedings of his State Society, which, for lack of room, we are obliged to greatly abridge.

Though this is the nineteenth session of the Kentucky State Medical Society, its first organization dates back to 1840, and was held in Frankfort. It however soon fell through, but was revived and chartered in 1851. There was no session held during the struggle for the Lost Cause, and until 1867, when it was convened and reorganized in Louisville, with Dr. D. N. Porter, as President. Since this reorganization the regular annual sessions have been held with continually increasing interest.

The society was called to order by President, Dr. Joseph W. Thompson, of Paducah, at noon, on the 7th of April, in the city of Shelbyville, with about forty members, mostly young men of the profession, present at the opening. Opening address by Dr. Lowry, of Shelbyville. Surgeon E. McLellan, U. S. A., read a lengthy and elaborate paper upon *Fibroid Tumours of the Uterus.* Dr. R. F. Logan, of Shelbyville, read a surgical report. Dr. L. P. Yandell, of Louisville, reported upon the *Medical Literature of Kentucky.* The Annual Address of the President was thoroughly practical and well received by his auditory. Dr. J. J. Speed, of Louisville, delivered the Annual Oration, reviewing ably the science of medicine. Dr. Richardson, of Louisville, read a valuable contribution upon the practice of medicine. Suitable resolutions were passed upon the announcement of the death of Professor Henry Miller, of Louisville.

The committee on the McDowell memorial reported that they had, in accordance with the instructions received from the society, organized, and asked that the society clothe them with plenary powers, which request was complied with, and the committee will devise means to soon erect the memorial.

Dr. A. B. Cook, of Louisville, having been expelled at a former session, was now reinstated in his membership by action of the body.

Dr. E. S. Gaillard, of Louisville, read an essay upon *Cerebro-spinal Meningitis.* The following essays were read and received, namely: On the *Medical Border Land,* by Dr. Andrew McFarland, of Illinois. Report of six cases *Ovariotomy,* four recoveries and two deaths; by Dr. D. W. Yandell, of Louisville; Report upon *Registration of Births, Deaths and Marriages*, by Dr. S. A. Foss, of Jefferson; Essay upon the *Diseases of the Conjunctiva*, by Dr. D. S. Reynolds, of Louisville; *Summer Complaints of Children*, by Dr. J. A. Larrabee, of Louisville; *Calculus in Females*, by Dr. R. H. Gale, of Owenton; *Albuminuria*, by Dr. Wm. Bailey, of Louisville; *Oxygen Gas*, by Dr. H. Wilson, of Louisville; *Glioma of the Retina*, by Dr. C. S. Fenner, of Louisville.

Dr. Lewis Rogers read the following resolutions, and moved in a few earnest words that they be adopted:

*Resolved*, That the State Medical Society of Kentucky does cordially unite with the American Medical Association in the memorial to Congress in support of a bill to increase the efficiency of the Medical Department of the United States Army, now before that honorable body.

*Resolved*, That this society considers it an act of justice that the members of so important a branch of the service, gentlemen of the highest professional attainments and excellence of character, and charged with such weighty and responsible duties, should hold the same relative rank and enjoy the same emoluments as members of the other staff corps of the army.

*Resolved*, That it be respectfully urged upon the members of Congress from the State of Kentucky to use their influence in the support of the bill in question.

The resolutions were unanimously adopted, and the secretary ordered to inform the Kentucky delegation in Congress of their adoption.

Dr. D. N. Porter offered the following resolutions, which were adopted:

*Resolved*, That the too common practice of physicians appearing in the public newspapers of the day can not be too strongly and unhesitatingly condemned as unbecoming our learned and honorable profession.

*Resolved*, That any member of the society who shall appear in a newspaper, *lagomica*, after this date, will expose himself to the danger of censure with the purpose of expulsion from the same for this non-professional and reprehensible practice.

*Resolved*, That physicians, as physicians, be kindly requested to write for Medical Journals only.

Dr. D. W. Yandell, of Louisville, presented a freshly amputated leg, removed by Esmarch's bloodless method.

With the usual resolutions of thanks, with the annual banquet, its champagne, wit and humor, with the smiles of fair women, with the joyous reunion of stout hearts around the social board, so closed the Nineteenth Annual Session of the Kentucky Society. B.

